# Suppurative parotitis and parapharyngeal space infection in an elderly patient – a Case report

**DOI:** 10.1186/1471-2334-14-S7-P66

**Published:** 2014-10-15

**Authors:** Lucian Chirilă, Cristian Rotaru, Iulian Filipov, Mihai Săndulescu

**Affiliations:** 1 “Dan Theodorescu” Clinical Hospital of Oral and Maxillo-Facial Surgery, Bucharest, Romania; 2 Carol Davila University of Medicine and Pharmacy, Bucharest, Romania; 3 Dental Concept Studio, Bucharest, Romania

## Background

The parotid glad region inflammatory processes can be specific or non-specific. Non-specific inflammations usually have a bacterial etiology, and are caused by the extension of a suppuration from a nearby region – the oral cavity or the nasopharynx. Other contributing factors can be the obstruction of the excretory duct and the salivary lithiasis.

## Case report

In this paper we present the case of an elderly woman with acute suppurative parotitis and parapharyngeal extension of the abscess. The patient had low hemoglobin levels, high uremic values and consequent tissue acidosis which determined an acute suppurative response. The patient underwent operative drainage of the abscess and removal of necrotic material. Intravenous antibiotics were also administered.

## Conclusion

Suppurations in the parotid region can disseminate in the parapharyngeal space, which can lead to a life-threatening infection in the absence of surgical treatment. High uremic values and tissue acidosis can determine a very extensive suppuration.

## Consent

Written informed consent was obtained from the patient for publication of this Case report and any accompanying images. A copy of the written consent is available for review by the Editor of this journal.

